# Creosote in Phthisis Pulmonalis

**Published:** 1899-03

**Authors:** L. H. Warner

**Affiliations:** Brooklyn, N. Y.


### CREOSOTE IN PHTHISIS PULMONALIS.

BY L. H. WARNER, M.D., BROOKLYN, N. Y.

After a brief review of creosote and guaiacol and the various methods of employing these products, the author says:

In the treatment of phthisis the administration of creosote causes the fever and cough to diminish and the patient to improve in appetite and flesh. On examination of the pulse it will be noted there is a smallness and rapidity indicating an increased anæmia produced by the powerful action of creosote. When creosote alone is used life is made more comfortable to the patient but it causes an earlier termination; if in combination with tonics, less anæmia is produced. It has antifermentative powers, and though it may not kill bacteria it destroys their ptomaines and renders their action non-toxic and inert. In the stomach of consumptives a pathological fermentation is at all times going on, and this process is overcome by the action of creosote. It takes oxygen from the blood, and is changed into carbolates and oxalates, as a result of oxidation, thus causing the blood to assume a deeper color. In the treatment of phthisis it becomes of special value if reinforced by nuclein. Nuclein increases the number of white blood corpuscles and is therefore a valuable agent in combating tuberculosis in its initial stage. Reviewing the aforementioned facts, we have creosote, guaiacol, nuclein and tonics as factors in the treatment of phthisis pulmonalis. How and in what proportion can they be best combined to become efficient in the treatment of this disease? Beef, milk and wheat peptonized, with cceosote and guaiacol, otherwise known as liquid peptonoids with creosote, is an eligible method of administering the above in combination. Each tablespoonful contains two minims of pure beechwood creosote and one minim of guaiacol combined with the nutrient and reconstituent properties of liquid peptonoids. In two different hospitals the entire consumptive wards were placed on this remedy with most excellent results, and it will be necessary to quote but a few of the many cases under observation:

Case 1.—M. P., female, aged 49, admitted to hospital June 2, 1898, family history tubercular. For some years patient has been troubled with severe attacks of cough, resulting from an attack of la grippe in 1894. Has dry hacking cough with gelatinous expectoration, containing bronchial alveolar epithelium in a state of fatty metamorphosis streaked with blood. Temperature 101 degrees. Loss of appetite and dyspeptic symptoms. Inspiration of cogwheel character, expiration high-pitched, and dullness on percussion. Patient has lost about thirty pounds within the last few months. Weighed on January 2d, 145 pounds. Blood count, forty-five per cent. Haem, 3,000,000 red cells, 7,500 white cells. Treatment began with one-tablespoonful doses of liquid peptonoids with creosote every four hours. Patient slowly improved, and on June 16th doses were doubled to two tablespoonfuls every four hours. Hereafter a rapid improvement took place. July 1st patient's cough had disappeared, no bacilli in sputum, appetite good, weight 151 pounds. This treatment was continued till July 26th, when patent left the hospital, apparently well. Weight 155 pounds; blood examination, Haem sixty-two per cent. red cells 3,650,000, white cells 7,200; no cough; good appetite.

Case 2.—E. W., male, age 20, family history tubercular, admitted June 9, 1898; hacking cough, purulent expectoration, temperature 100 degrees, night sweats, loss of appetite and weight; blood examination, forty-three per cent. Haem, 2,700,-000 red cells, 7,000 white cells; weight ninety-eight pounds; examination of sputum, bronchial and alveolar epithelium, bacilli. Same treatment as in case 1, began June 9th; patient improved. June 26th coughed but little; no bacilli in sputum; appetite good; weight 103 pounds. July 13th discharged, apparently well; no cough, no night sweats, appetite ravenous, weight 105 pounds, blood count Haem sixty-one per cent, red cells 3,600,000, white cells 6,800.

All teburcular cases under my observation improved under this treatment, while others under the plain doses of creosote gtt v to xx showed but little improvement.

---

### AN UNCONSCIOUS ACCOUCHEMENT.

There is recorded in *Medecine moderne* for August 31, a case of accouchement in which the mother appears to have been unconscious of her delivery. The pains, which had been regular, gradually grew fewer, and the doctor retired giving instructions that he was to be summoned about two hours later. On his return, the patient declared that the pains were strong, but the midwife assured the doctor that they were not strong enough to justify the anticipation of a speedy accouchement. The doctor caused the patient to be uncovered, when great was his astonishment to find the child lying between the mother's thighs, not breathing and motionless, the head plunged in a flood of amniotic fluid. A hot bath and alcohol friction to the spine recalled the infant to life. The child weighed somewhat under seven pounds. This recital is not without interest from a medico-legal point of view.—*New York Medical Journal.*

